# Regional Odontodysplasia with Generalised Enamel Defect

**DOI:** 10.1155/2016/4574673

**Published:** 2016-12-21

**Authors:** A. M. Al-Mullahi, K. J. Toumba

**Affiliations:** ^1^Oral Health Department, Sultan Qaboos University Hospital, Sultan Qaboos University, Muscat, Oman; ^2^Paediatric Dentistry, Leeds Dental Institute, University of Leeds, Leeds, UK

## Abstract

Regional odontodysplasia (ROD) is uncommon developmental anomaly, which tends to be localised and involves the ectodermal and mesodermal tooth components. A five-year-old female was referred to Department of Child Dental Health at the Leeds Dental Institute regarding malformed primary teeth. On examination 64, 74, and 72 had localised hypomineralized enamel defect. The crown of 55 was broken down with only the root remaining below the gingival level. 54 has a yellowish brown discolouration with rough irregular surface. The upper anterior teeth show mild enamel opacity. Radiographically, 55 and 54 had thin radioopaque contour, showing poor distinction between the enamel and dentine and the classic feature of a wide pulp chamber. 15, 16, and 17 were developmentally delayed and were displaying the characteristic “ghost appearance.” Comprehensive dental care was done under local anaesthesia and it included extraction of the primary molars affected by ROD, stainless steel crown on 64, and caries prevention program. Fifteen months following the initial assessment the patient's oral condition remains stable and she is under regular follow-up at the department. Paediatric dentists should be aware of this anomaly as it involves both dentitions and usually requires multidisciplinary care.

## 1. Introduction

Regional odontodysplasia (ROD) is uncommon developmental anomaly, which tends to be localised and involves the ectodermal and mesodermal tooth components [1]. This anomaly has no reported association with any specific racial group; however female is slightly more affected than male at ratio of 1.4 : 1 [1]. Although this anomaly usually affects dental tissues, case reports have documented the presence of regional odontodysplasia with epidermal nevus syndrome [2, 3], hypoplasia of the affected side of the face [4], hypophosphatasia [5], hydrocephalus and mental retardation [6], and ipsilateral vascular nevi [4, 7]. The etiology remains uncertain of many possible causes that have been proposed in the literature. This included somatic mutation of the dental lamina [8], viral infection [9], medication taken during pregnancy [10], local circulating disorder like vascular nevi on the skin of the affected side of the face [11, 12], failure of migration, and differentiation of neural crest cells [2].

Regional odontodysplasia typically affects one quadrant of the jaw, although it does occasionally cross the midline [7, 13–15]; however there are few cases where this anomaly has affected either the maxillary and mandibular quadrants of the same side [16, 17] or both quadrants of the same jaw [16, 18] and it is extremely rare for the anomaly to affect all four quadrants [13, 19]. Affected teeth are usually in a continuous series, although it may skip a tooth or a group of teeth [12]. Primary teeth affected by this anomaly are usually followed by affected permanent successors; however it is very rare to find normal permanent teeth to follow affected primary ones [20]. Patients with regional odontodysplasia usually present with pain and dental abscess formation [21–23] which can be seen even in the absence of dental caries [24]. Other presenting complaints include delay or failure of eruption [14, 25], gingival swelling [26, 27], and abnormal clinical appearance of the teeth [1]. Clinically, affected teeth are usually smaller than normal with a rough surface texture and extensive pits and grooves. The enamel is hypocalcified and/or hypoplastic with a yellow or brown discolouration and can be soft on exploration with a dental probe [27]. Affected teeth are more susceptible to dental caries due to defective mineralisation [28, 29]. Radiographically, teeth with ROD have shortened roots with open apices [30]. The distinctive “ghost appearance” of these teeth is due to the wide pulp chamber [1, 22, 24] and the reduced thickness of enamel and dentine with loss of demarcation between these tissues.

In this report, we describe a case of regional odontodysplasia with generalised enamel defects in the primary dentition.

## 2. Case Report

A 5-year-old Caucasian female attended the consultant clinic at Paediatric Dental Department of Leeds Dental Institute, following a referral by her general dental practitioner regarding malformed deciduous teeth. The child's chief complaint on presentation was pain from the teeth in upper right quadrant. The pain started one week ago; it was associated with eating and it lasted for a short period. She did not suffer from any dental infection or associated facial swelling. The patient's perinatal and medical history was noncontributory and the mother reported no previous family history of dental anomalies.

### 2.1. Clinical Examination

**Figure 1:**
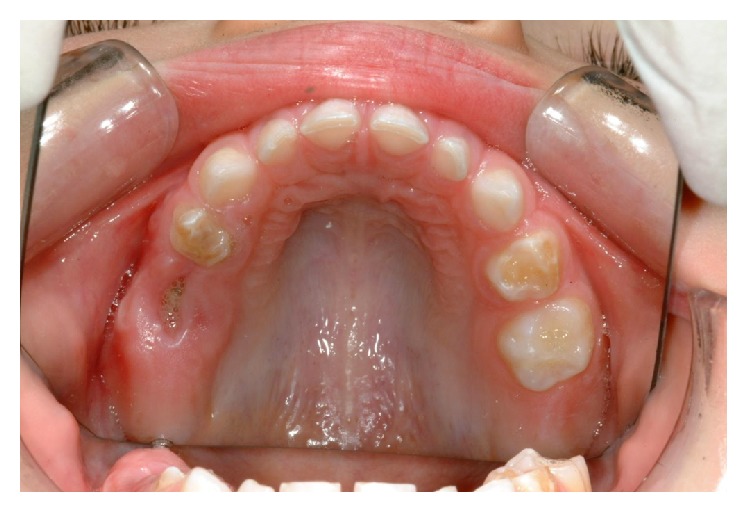
Broken down 55 to subgingival level and hypomineralization and hypoplasia of 54 and 64.

**Figure 2:**
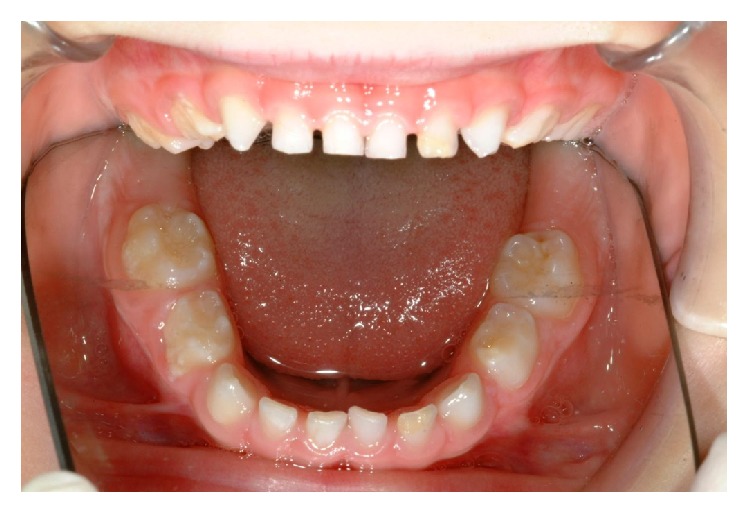
The lower arch.

**Figure 3:**
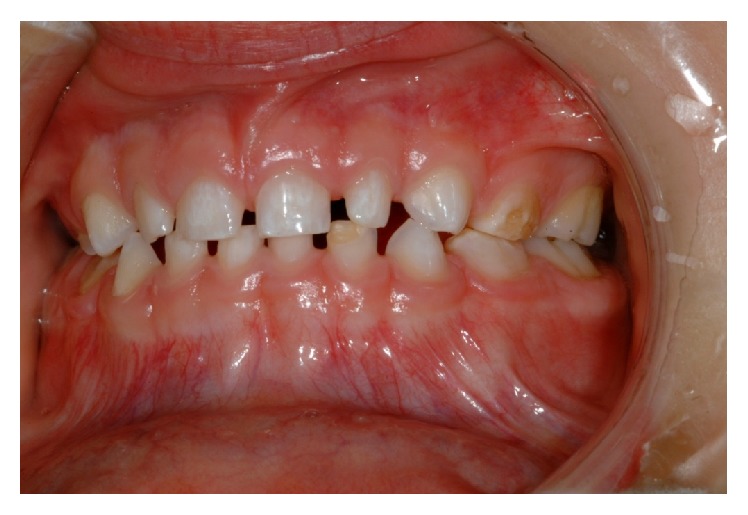
Photograph showing the enamel hypomineralization affecting the labial surface of the upper anterior teeth, hypoplasia in 72, and hypomineralization in 64 and 74.

Extraoral examination revealed no pathological features. Intraorally the mucosa had normal colour and texture. In the maxillary arch, the first primary molars had hypomineralized and hypoplastic enamel defects with 54 more severely affected with rough and irregular surface, which had a yellow brown discolouration (Figure 1). The crown of 55 was broken down with only the root remaining below the gingival level. The upper incisors had mild enamel opacity on the labial surfaces and 53 showed enamel pitting (Figure 2). The mandibular arch had a normal number of teeth and the primary molars appear more yellowish in colour (Figure 3). The mesial marginal ridge of 74 had localised enamel opacity and the incisal half of the 72's labial surface had a hypoplastic defect.

### 2.2. Radiographic Examination

**Figure 4:**
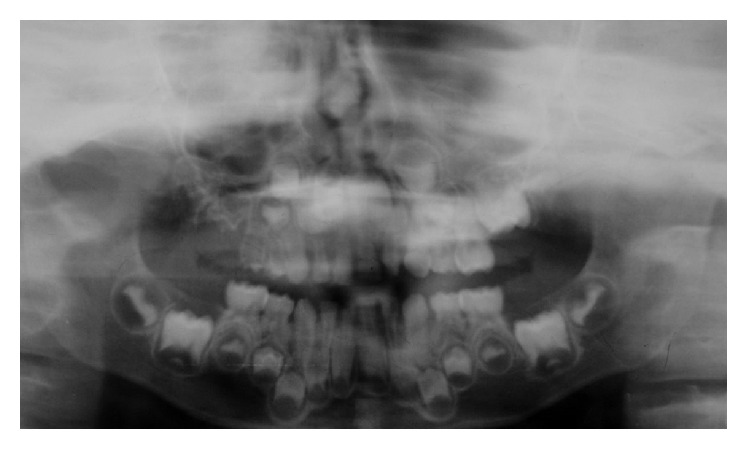
OPG radiograph.

The patient's general dental practitioner sent an orthopantomogram (OPG) radiograph (Figure 4) and we have taken bitewing radiographs for caries assessment and diagnosis. The radiographic image showed 55 and 54 with classical radiographic features of “ghost teeth,” with a thin radioopaque contour, showing poor distinction between the enamel and dentine and wide pulp chamber. 55 had very thin retained roots with complete loss of the crown. 15, 16, and 17 are developmentally delayed in relation to the corresponding teeth on the contralateral side of the arch and displaying the characteristic features seen in teeth with regional odontodysplasia. Although 54 is affected by ROD, the permanent successor appears to be in the same developing stage as the other first premolars. Based on the clinical and radiographic findings, our diagnosis was regional odontodysplasia and generalised enamel defects.

### 2.3. Treatment

**Figure 5:**
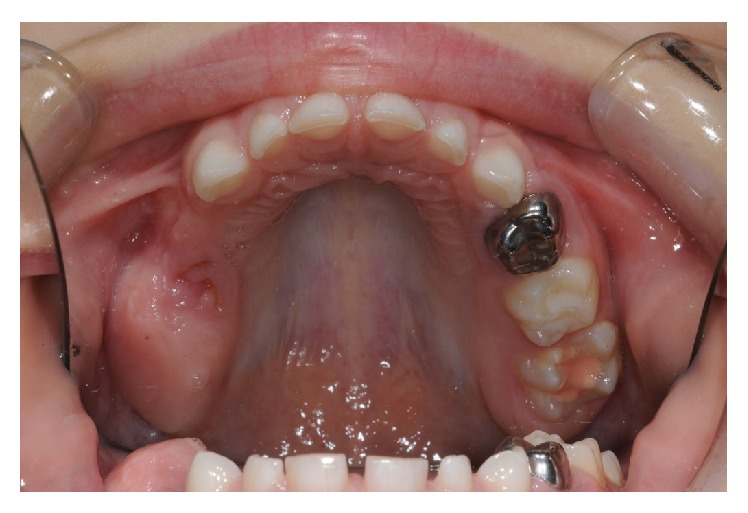
Upper arch, posttreatment.

**Figure 6:**
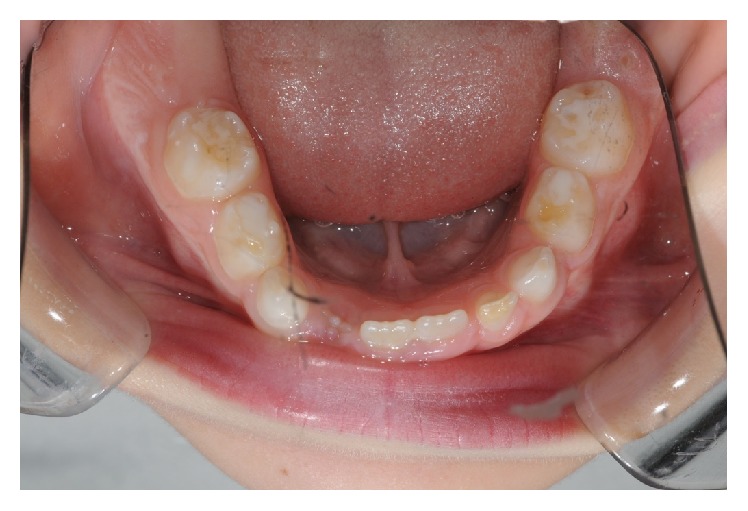
Lower arch, posttreatment.

The initial treatment plan included extraction of 55, stainless steel restoration of 54 and 64 under local anaesthesia, and caries preventative program that included regular fluoride varnish, casein phosphopeptides and amorphous calcium phosphate applications, and fissure sealant of the primary molars. However, on a subsequent visit, 54 become infected and a draining sinus developed buccal to that tooth. Hence, the treatment plan has changed to include extraction of 54 and removal of the remaining roots of 55 under local anaesthesia. The planned treatment was done under local anaesthesia over several visits (Figures 5 and 6). The removal of the remaining roots of 55 was not possible without raising a flab and bone removal because of the thin roots. Hence, the clinical decision was to remove the superficial part and leave the remaining part of the root as it is not infected. Extracted 54 and remaining roots of 55 were sent for histopathological examination. Following the eruption of the upper left first permanent molar there was deep fissure with area of enamel opacity on the mesial aspect of the palatal wall. Hence, the tooth was fissure sealed using Fuji Triage™ to prevent plaque accumulation.

### 2.4. Histology Report

The histopathology report for the sent specimen (54 and remaining roots of 55) described malformed enamel with underlying irregular, poorly mineralised dysplastic dentine. The pulp shows nonfusion of one pulp horn. The center of the pulp shows necrotic material with inflammatory cells associated with mineralisation. The report confirmed the clinical and radiographic diagnosis of regional odontodysplasia.

### 2.5. Follow-Up

Fifteen months following the initial assessment the patient's oral condition remains stable with no evidence of dental disease. The patient was placed on a regular follow-up schedule at the department to monitor the eruption of the teeth affected by this anomaly and the presence of mineralisation defects in the remaining permanent teeth and assessing its severity to consider the treatment options and future dental care.

## 3. Discussion

The presented case shares many features abundantly described in patients with regional odontodysplasia; these include slight gender tendency toward female [1] and unilateral involvement of maxilla which is twice as common to mandibular involvement [1]. However, there are few features which are rarely reported like radiographic evidence of a normally developing permanent successor (upper right first premolar) to follow affected primary predecessor (upper right first primary molar) [20] or the generalised enamel hypomineralization and hypoplasia affecting the other primary teeth which have not been reported in any case of regional odontodysplasia. The enamel defects seen in teeth with ROD are usually severe [13, 27]; however the remaining teeth usually have normal enamel and dentine [1]. In this case, the severity and pattern of mineralisation defects seen in the unaffected teeth by ROD, mineralization of the permanent successors, size of the pulp, and radiographic features do not conform with the diagnosis of ROD. These defects are likely to be developmental defect of enamel (DDE) in the primary dentition. The DDE in primary teeth are relatively common with prevalence ranging from 8.4% to 48.0% [31, 32] and in primary teeth of healthy children in developed countries ranging from 24% to 49% [33, 34]. Many risk factors have been associated with the development of DDE and it includes medical conditions [35, 36], social factors [37], medical problems during pregnancy [38], absence of breast feeding [38], nutritional problems [37], and mutation in the amino acid sequence of amelogenin gene [39]. The high prevalence of developmental defect of enamel in the primary dentition, the diversity of the risk factors, and clinical presentation favour the diagnosis of DDE.

Treatment of ROD remains a clinical dilemma as it is controversial with lack of consensuses in managing this anomaly. Early extraction of the affected teeth has been proposed by many authors [13, 16, 22, 27] as these teeth might develop dental pathology even in the absence of dental caries due to the thin enamel layer and the presence of enamel and dentinal cleft which allow ingress of microorganism to the dental pulp [1]. In addition, the defective mineralisation of the involved teeth results in undesirable appearance and poor dental aesthetics. The extraction was followed in some cases by prosthetic replacement [40, 41]. Some authors have argued for maintaining the noninfected affected teeth to allow normal jaw development and reduce the risk of psychological trauma associated with premature tooth loss [24, 27]. Other treatment approaches in ROD include coverage restorations [25] and autotransplantation of teeth in the permanent dentition [20]. The management of ROD involves interventional dental care in both dentitions and it requires multidisciplinary care. The present case was managed by extraction of the affected teeth which is in agreement with previous reports in the literature [16] and stabilising the remaining primary teeth. The patient is under regular follow-up in the department to assess the developing permanent dentition affected by ROD and to determine the extent of involvement and severity of the generalised enamel hypomineralization and plan future dental care accordingly.

## 4. Conclusion 

ROD in the primary dentition can be easily mistaken for grossly carious teeth. However early diagnosis of this condition is important as it involves both dentitions and usually requires multidisciplinary care.
